# Exploring the psychometric properties of the Working Alliance Inventory in general practice: a cross-sectional study

**DOI:** 10.3399/bjgpopen20X101131

**Published:** 2020-12-16

**Authors:** Liesbeth Hunik, Shelley Galvin, Tim olde Hartman, Elizabeth Rieger, Peter Lucassen, Kirsty Douglas, Pauline Boeckxstaens, Elizabeth Sturgiss

**Affiliations:** 1 Department of Primary and Community Care, Radboud University, Nijmegen, The Netherlands; 2 UNC Health Sciences at MAHEC, Mountain Area Health Education Center, Asheville, USA; 3 Research School of Psychology, Australian National University Research School of Psychology, The Australian National University, Acton, Australia; 4 Australia Academic Unit of General Practice, Australian National University Medical School, The Australian National University, Garran, Australia; 5 Department of Family Medicine and Primary Healthcare, Ghent University, Gent, Belgium; 6 Department of General Practice, Monash University, Melbourne, Australia

**Keywords:** physician—patient relations, primary health care, quality of health care

## Abstract

**Background:**

The therapeutic alliance is a framework from psychology that describes three components: goals, tasks, and bond. The Working Alliance Inventory adapted for general practice (WAI-GP) measures the strength of the therapeutic alliance between the patient and the clinician, and it could be useful in both research and clinical settings.

**Aim:**

To determine if the patient score on WAI-GP can delineate the three components (goals, tasks, and bond), and to test concurrent validity with the Consultation and Relational Empathy (CARE) measure and the Patient Perception of Patient-Centredness (PPPC) measure.

**Design & setting:**

A cross-sectional study took place in 12 general practice waiting rooms in Australia.

**Method:**

The research instruments included the 12-item WAI-GP (the patient version), the CARE and PPPC measures, plus a survey of demographics and reason for consultation. To perform a principal components factor analysis of the WAI-GP, this dataset was combined with an existing dataset. The Spearman rank correlation was used to determine concurrent validity between the WAI-GP and the CARE and PPPC measures.

**Results:**

Participants (97–99%) reported a strong positive alliance after the consultation (average WAI-GP mean 4.27 ± 0.67 out of 5, *n* = 146). Factor analysis could not separate the three components (one factor, eigenvalue >1; Cronbach’s α = 0.957; *n* = 281). Concurrent validity was supported by moderate correlations with the other measures (PPPC *ρ* = –0.51, *P*<0.005, CARE *ρ* = 0.56, *P*<0.005).

**Conclusion:**

Three components could not be identified, but the WAI-GP has a high internal consistency and concurrent validity with moderate correlations with the CARE and PPPC. A more diverse sample may better distinguish the three components leading to more specific feedback to clinicians on their consultation practices.

## How this fits in

The doctor–patient relationship depends on a variety of components, including trust, empathy, shared decision making, and patient-centredness, and currently multiple tools are needed to measure different parts of the therapeutic alliance. The WAI-GP is an instrument that divides the therapeutic alliance into three components: goals, tasks, and bond. It was found that the WAI-GP is correlated with measures used for empathy, patient-centredness, shared decision making, and depth of relationship. Therefore, it could be used as a more comprehensive and concise measure for relational aspects of primary care. It may be helpful within research, teaching, and clinical settings to provide feedback to clinicians on the strength of the therapeutic alliance within their consultations.

## Introduction

There is ever more evidence that a high-quality doctor–patient relationship has a beneficial effect on patient outcomes.^1–4^ In primary care, much research has been conducted on the doctor—patient relationship,^5–9^ and results support that positive therapeutic relationships improve health and symptom outcomes for patients.^10^ The authors' international team is working towards a measure for the doctor—patient relationship that can be used in different healthcare settings to improve primary care interventions.^11^ This article describes the next step in the journey.

The doctor–patient relationship depends on a variety of components, including trust,^12,13^ empathy,^14^ shared decision making,^15^ and patient-centredness.^10^ Multiple instruments have been developed to measure the different components of the doctor–patient relationship; for example, the CARE measure and the PPPC measure. The CARE is a widely used validated questionnaire for measuring the empathy of the doctor as experienced by the patient.^14^ The PPPC measure is a validated tool for quantifying the patient’s perspective of the patient-centredness of a consultation.^16^

However, there is not yet a measure that encompasses all components of the relationship between patients and doctors in primary care,^7,8^ and using multiple surveys in implementation trials is not feasible. A single tool is being sought that can measure multiple aspects of the doctor–patient relationship in primary care to be used in research, teaching, and clinical practice across different healthcare settings.

The debate about the effectiveness of key elements of the doctor—patient relationship has directed attention to another theoretical framework, namely the therapeutic alliance.^9^ Originating from the discipline of psychology, Bordin developed a now well-researched framework on the therapeutic alliance, which he termed the 'working alliance'.^17^ The working alliance is a framework that includes three components: collaborative goals or target outcomes that are mutually agreed on by the healthcare practitioner and the patient; the tasks or steps the doctor or patient must take to achieve their goals; and the bond between doctor and patient.^17^

Bordin’s tripartite conceptualisation of the therapeutic relationship has been used to develop the Working Alliance Inventory (WAI).^18^ This questionnaire was originally delivered to both patient and practitioner to measure goal setting, tasks, and bond from the patient’s and practitioner’s perspective. The tripartite concept provides more detail of the consultation and can, therefore, be used to give tailored feedback on those parts.^19^ The WAI has been used in research in several countries for medical care, including in Canadian community primary care^20^ and in secondary care in the Netherlands.^21^ Higher scores on the WAI are associated with better healthcare outcomes.^19,22,23^

In a recent pilot study, the WAI was adapted for Australian general practice — the WAI-General Practice (WAI-GP)^24^ — and was found to have concurrent validity with a measure of shared decision making and the depth of the doctor–patient relationship. It was reassuring to see that the measure was not influenced by social desirability.^24^ As is seen in other studies comparing doctor and patient assessment of relationship and communication.^9,25–29^ The WAI-GP scores from the patients and the family doctors were not similar.^24^ Thus, the perspective of the patient and doctor should be assessed independently depending on the research question and their scores should not be amalgamated, as is typically done in psychology.^18^

The study seeks to add to understanding of the WAI-GP by exploring its ability to reflect the three parts of the therapeutic alliance as stipulated by Bordin, from the patient’s perspective. In addition, the study further tests the concurrent validity of the WAI-GP with other commonly used tools for exploring the therapeutic relationship — the CARE and PPPC — to strengthen the evidence for its use in primary care.

## Method

For this cross-sectional survey study, adult patients were recruited who attended five general practices. These practices had an existing relationship with the authors' academic department through teaching or research. Recruitment occurred in two steps. First, from February until May 2019, practices were recruited via email from a list of teaching practices. The second step was to recruit the patients in the practice waiting room. When patients came in for an appointment with their family doctor, a research assistant (LH) invited patients to participate in the study. All patients were invited to participate but a record was not kept of those who declined. The patients completed the surveys without assistance.

The participants completed the questionnaires (Supplementary Appendix 1) both before and after their appointment with their GP. Before the appointment, patients were asked to fill in demographic information (two items), current health status (four items), whether they were seeing their preferred GP, the reason for consultation, how long they had known the GP, and if they were attending with a support person.

After the appointment, they filled in the WAI-GP, the CARE and the PPPC instruments, and details of the quality of the consultation (for example, did the doctor listen carefully? Did they show respect? Did they spend enough time with you?). The WAI-GP questionnaire has 12 items measured on a five-point Likert scale, ranging from ‘strongly agree’(1) to ‘strongly disagree’ (5). The total score is the average of the 12 questions for a maximum score of five (Supplementary file 1). The CARE measures the empathy of the physician as experienced by the patient^30^ and contains 10 questions with a five-point Likert scale ranging from ‘poor’(1) to 'excellent' (5) (there is also the option of 'does not apply' ). The PPPC tool measures patient perceptions of patient-centredness using nine questions, with a four-point Likert scale ranging from ‘completely' (1) to ‘not at all’ (4).

### Analysis

#### Descriptive statistics

For the descriptive analysis, only surveys with the WAI-GP completed were included (that is, no missing items on the WAI-GP). The analysis contained descriptive statistics for demographics, health status, details about the doctor–patient relationship, and details about the quality of consultation with the GP. The demographics were compared with the Bettering the Evaluation and Care of Health (BEACH) dataset, to see if the data were comparable with the Australian general practice patient population.^31^ The questions about the quality of the consultation were compared with the Patient Experiences Survey from the Australian Bureau of Statistics data to compare the experience of this consultation with the national dataset.^32^

#### Concurrent validity

The concurrent validity between the WAI-GP and the CARE and PPPC measures were assessed using Spearman *ρ* correlations for non-normally distributed variables. Spearman *ρ* correlations were used because the data were positively skewed, as also occurred in the pilot study.^24^ For questionnaires with missing data, participants were only included who completed ≥11 items of 12 of the WAI-GP, ≥8 of 10 items of the CARE,^33^ and ≥8 of 9 items of the PPPC.^16^ It was hypothesised that the WAI-GP scores would be correlated with the CARE and PPPC scores. (Spearman: weak relationships *ρ* = 0.01–0.34; moderate relationships *ρ* = 0.35–0.64; strong relationships *ρ* = 0.65–1.00.)

#### Factor analysis

Factor analysis is used to group questions within a survey to explore any relationships between them.^34^ For the factor analysis, this dataset was combined with that from the original pilot study. This pilot study contained 139 patients, recruited from seven general practices^24^ and used the same sampling technique as this study. Participants were only included who had complete responses for the WAI-GP (all 12 items). A sample of 300 participants was determined to be adequate for the factor analysis, according to Yong and Pearce.^33^

The internal reliability of the combined dataset was assessed with Cronbach’s alpha. The factor analysis used a principal components analysis with extraction criteria of eigenvalue >1 and oblique rotation. Data analyses were conducted using SPSS (version 24) with statistical significance at *P*<0.05.

## Results

### Demographics

**Figure 1.**
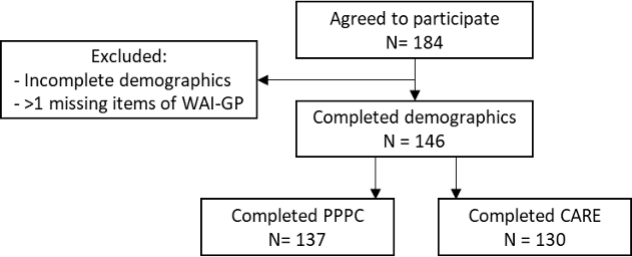
Flowchart for concurrent validity analyses. WAI-GP = Working Alliance Inventory for General Practice. PPPC = Patient Perception of Patient-Centredness. CARE = Consultation and Relational Empathy (CARE)

From the 184 surveys that were collected, 146 (79.3%) had complete data for all demographics and the WAI-GP (Figure 1). Ninety-one (62.3%) of the participants were female, most of the patients were aged ≥45 years (52.1%). Ninety-eight (67.1%) of the patients reported a chronic condition (Table 1). Most of the patients (73.3%) were seeing their preferred GP that day (Table 2). The data for this study were comparable with the BEACH sample.^31^ Participants reported a strong quality of the consultation in the survey when compared with the National Patient Experiences Survey.^32^

**Table 1.** Participant characteristics compared with Australian general practice patient population

Patient characteristics	*n* (%)	BEACH 2015–2016
**Sex**		
Male	55 (37.7)	43.4 %
Female	91 (62.3)	56.6%
**Age (years)**		
18–24	25 (17.1)	19.2% <25 years
25–34	29 (19.9)	22.8% 25–44 years
35–44	16 (11.0)	
45–54	18 (12.3)	27.2% 45–64 years
55–64	18 (12.3)	
65–74	24 (16.4)	14.7%
75–84	12 (8.2)	16.0% >75 years
≥85	4 (2.7)	
Chronic illness	98 (67.1)	

**Table 2.** Patients' consultation experiences and reasons for consultations

**Patient characteristics**	***n* (%**)	**NPES** ^a^
**Preferred GP**		
Yes	107 (73.3)	
No	18 (12.3)	
Prefer not to say or no response	21 (14.4)	
**How long known the GP**		
First meeting today	19 (13.0)	
Second appointment	16 (11.0)	
<1 year	25 (17.1)	
About 1–5 years	50 (34.2)	
>5 years	32 (21.9)	
Prefer not to say	2 (1.4)	
No response	2 (1.4)	
**Reasons for consultation** ^b^		
Find out what’s wrong or diagnosis	43 (29.5)	
For reassurance	9 (6.2)	
Get results or investigations	34 (23.3)	
Treatment, prescriptions, or procedures	46 (31.5)	
Routine check	20 (13.7)	
Review	18 (12.3)	
Ask for a referral	17 (11.6)	
**Attending the appointment with**		74%^a^
Alone	125 (85.6)	
With a support person	19 (13)	
Prefer not to say	1 (0.7)	
No response	1 (0.7)	
**Did GP listen carefully?**		81%^a^
Yes	144 (98.6)	
No	0	
Prefer not to say	2 (1.4)	
**Did GP spend enough time?**		76%^a^
Yes	142 (97.3)	
No	2 (1.4)	
Prefer not to say	2 (1.4)	
**Was consultation about chronic condition?**	46 (31.5)	
Yes	72 (49.3)	
No, the consultation was not about a chronic condition	21 (14.4)	
I don’t have a chronic condition	7 (4.8)	
Prefer not to say	0	

^a^2017–18 National Patient Experiences Survey, Australian Bureau of Statistics.^28,31^ ^b^More than one choice permitted.

### Concurrent validity

The WAI-GP was completed by 146 participants (mean 4.27 ± 0.67), the PPPC by 137 (mean 1.42 ± 0.46), and the CARE by 130 participants (mean 4.58 ± 0.68) (Table 3). All three measures were highly positive, correlating with a good consultation experience and strong therapeutic alliance. The patient version of the WAI-GP was moderately correlated with the CARE (*ρ* = 0.563, *P*<0.005) and PPPC (*ρ* = -0.508, *P*<0.005).

**Table 3.** Results of WAI-GP and other scale scores

**Survey**	**Response options**	**Mean ±** **SD**
WAI-GP (*n* = 146)	Strongly agree (1) to strongly disagree (5)	4.27 ± 0.67
Goal		4.25 ± 0.69
Bond		4.22 ± 0.69
Task		4.36 ± 0.71
PPPC (*n* = 137)	Completely (1) to not at all (4)	1.42 ± 0.46
CARE (*n* = 130)	Poor (1) to excellent (5)	4.58 ± 0.68
WAI-GP pilot study (*n* = 139)		4.33 ± 0.56
Combined (*n* = 281)		4.30 ± 0.62

WAI-GP = Working Alliance Inventory for General Practice; PPPC = Patient Perception of Patient-Centredness; CARE = Consultation and Relational Empathy

### Combined studies: factor analysis

**Figure 2.**
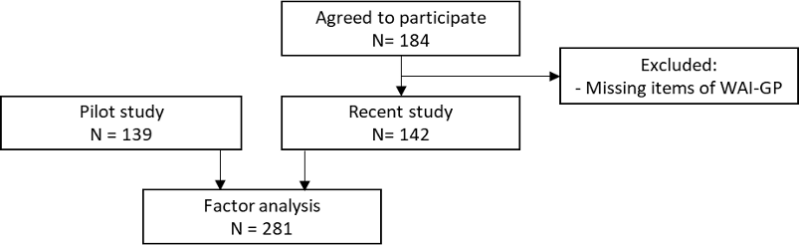
Flowchart for factor analysis. WAI-GP = Working Alliance Inventory for General Practice

Combining the pilot data with the current data resulted in a total of 281 participants (Figure 2). The WAI data for this study (*n* = 146) are highly positively skewed (mean 4.27 ± 0.67). In the pilot study, 139 participants completed the WAI-GP, with a mean 4.33 (standard deviation ±0.59) (Table 3), the data were also positively skewed.^24^ The patients reported a strong positive experience, with the GP listening carefully and the GP taking enough time (Table 2).^31^

Using principal components factor analysis, the three domains were not found (that is, goals, tasks, and bond) in the analysis. One overall alliance factor with eigenvalue >1 was identified. The interrelationships of the 12 items was moderate 0.482–0.625, *P*<0.001. When exploring the internal consistency, a high internal reliability was found (Cronbach’s alpha = 0.957).

## Discussion

### Summary

This study explored whether the results of the WAI-GP patient survey align with the three components of Bordin’s therapeutic alliance framework (goal, task-, bond) within the patient’s perspective of the alliance. Despite a sample of 281 patients, only one factor could be identified with a high internal consistency. Almost all participants (97–99%) reported a strong quality of the consultation in the survey, compared with 74–81% of the participants in the National Patient Experiences Survey.^32^ It was found that the WAI-GP questionnaire was moderately correlated with the CARE and PPPC.

The moderate correlation with the CARE and PPPC was expected, given these questionnaires measure empathy and patient-centredness, which are parts of the therapeutic alliance. In the pilot study, the WAI-GP was found to have concurrent validity with a measure of shared decision making and the depth of the doctor–patient relationship.^24^ This means that the different parts of the relationship from the patient’s perspective (for example, empathy, patient-centredness, shared decision making) can be measured within the WAI-GP, thus the WAI can be used as a more complete and concise measure for the alliance in place of other validated and often used questionnaires.

Possible explanations for these results include that the data were too positively skewed to detect the separate components, or that the WAI-GP cannot delineate the three individual components of alliance. Another explanation can be that the different components of the therapeutic alliance influence each other and, therefore, make it more difficult to be separately measured with the WAI-GP. This aligns with the theory of Bordin, because the three components need to be simultaneously strong to have an effective alliance.^17,18,21^

### Strengths and limitations

A sample size of 300 participants was selected, while a sample of 281 was reached (when the two datasets were combined). Based on recommendations, a sample of 281 is well within the appropriate sample size range.^35^ Looking at the characteristics of the sample, the participant sample corresponds with the national Australian dataset of patients in general practices.^31^ This suggests that the findings are more likely to be applicable across the Australian general practice population. Two datasets were combined, with a lot of similarities.

Combining this data, there were no differences in the sampling or the administration, the participants were similar. Demographics showed only a small difference between patients having a chronic disease (67.1 compared with 50.7), the sample came from multiple clinics from two different states in Australia. While pooling data were used across the two administrations, some bias may have entered into the results.^36^ Both these samples were written surveys, it is possible that sampling from an online population about their most recent clinical experience may give a wider spread of results.^37^ Another explanation is that the 20% of patients with incomplete surveys may have had a different consultation experience to those who completed their surveys. In future research, it would be interesting to see if an online sample would result in a broader range and a less positively skewed sample, for the principal components factor analysis to delineate the three factors.

### Comparison with existing literature

There are mixed findings in the literature about the number of factors in the WAI, with previous research indicating considerable overlap between the three components.^19^ Another version of the WAI was shortened from the original longer version, the Working Alliance Inventory-Short Revised (WAI-SR),^36^ and a subsequent language translation also retained a three-factor structure.^37^ However, when the WAI-SR was modified for use in career counselling^38^ and offender rehabilitation,^39^ only one overarching alliance factor was found. Likewise, with the authors' modification for use in primary care, only one factor was identified.

In previous studies, a high score on the WAI has been found to be strongly associated with better patient outcomes in psychotherapy^19,22^ and counselling.^17,39^ The authors set out to complete this study because it was hypothesised that if the three separate components of the alliance could be delineated, then more specific feedback could be given to clinicians on how to improve their therapeutic alliance with patients. For example, if goal setting is scored more weakly than bond, clinicians could be assisted with this part of the consultation to improve their overall effectiveness with patients.

### Implications for research and practice

For this study, the dataset was combined with that from the pilot study to give an acceptable sample size for the principal components factor analysis. However, the participants in the sample reported a very strong quality of the consultation. As a result, the sample did not have a broad range of scores. It would be interesting to conduct a factor analysis with consultations with a broader range of WAI-GP scores, including some patients who perceived a less strong alliance. It is important to consider how the survey would perform in those with a weaker alliance and this could provide helpful feedback to clinicians for clinical practice improvement.

The therapeutic alliance theoretical framework is a useful construct for understanding the doctor–patient interaction. To assist clinicians to improve their therapeutic relationship with patients, the WAI-GP could be used as a specific, reliable, and valid measure of the alliance. The WAI-GP is correlated with measure of empathy, patient-centredness, shared decision making,^24^ and depth of relationship.^24^ Therefore, compared to currently available tools, it is a more comprehensive and concise measure for relational aspects of primary care.

## References

[1] Conboy LA, Macklin E, Kelley J (2010). Which patients improve: characteristics increasing sensitivity to a supportive patient—practitioner relationship. Soc Sci Med.

[2] Kelley JM, Kraft-Todd G, Schapira L (2014). The influence of the patient—clinician relationship on healthcare outcomes: a systematic review and meta-analysis of randomized controlled trials. PLoS One.

[3] Starfield B, Wray C, Hess K (1981). The influence of patient—practitioner agreement on outcome of care. Am J Public Health.

[4] Kearley KE, Freeman GK, Heath A (2001). An exploration of the value of the personal doctor—patient relationship in general practice. Br J Gen Pract.

[5] Jones DE, Carson KA, Bleich SN, Cooper LA (2012). Patient trust in physicians and adoption of lifestyle behaviors to control high blood pressure. Patient Educ Couns.

[6] Berry LL, Parish JT, Janakiraman R (2008). Patients' commitment to their primary physician and why it matters. Ann Fam Med.

[7] Greenhalgh T, Heath I (2010). Measuring quality in the therapeutic relationship — part 1: objective approaches. Qual Saf Health Care.

[8] Ridd M, Shaw A, Lewis G, Salisbury C (2009). The patient—doctor relationship: a synthesis of the qualitative literature on patients' perspectives. Br J Gen Pract.

[9] Stewart M, Brown JB, Donner A (2000). The impact of patient-centered care on outcomes. J Fam Pract.

[10] Boeckxstaens P, Meskens A, Van der Poorten A (2020). Exploring the therapeutic alliance in Belgian family medicine and its association with doctor—patient characteristics: a cross-sectional survey study. BMJ Open.

[11] Hall MA, Dugan E, Zheng B, Mishra AK (2001). Trust in physicians and medical institutions: what is it, can it be measured, and does it matter?. Milbank Q.

[12] Pearson SD, Raeke LH (2000). Patients' trust in physicians: many theories, few measures, and little data. J Gen Intern Med.

[13] Mercer SW, McConnachie A, Maxwell M (2005). Relevance and practical use of the consultation and relational empathy (care) measure in general practice. Fam Pract.

[14] Dillon EC, Stults CD, Wilson C (2017). An evaluation of two interventions to enhance patient—physician communication using the observer OPTION^^5^^ measure of shared decision making. Patient Educ Couns.

[15] Stewart M, Meredith L, Ryan BL, Brown JB (2004). The patient perception of patient-centeredness questionnaire (PPPC).

[16] Bordin ES (1979). The generalizability of the psychoanalytic concept of the working alliance. Psychotherapy: Theory, Research & Practice.

[17] Horvath AO, Greenberg LS (1989). Development and validation of the Working Alliance Inventory. J Couns Psychol.

[18] Elvins R, Green J (2008). The conceptualization and measurement of therapeutic alliance: an empirical review. Clin Psychol Rev.

[19] Chan AD (2008). The working alliance as a conceptual framework of patient-centredness: The development of the primary care Working Alliance Inventory. [Doctoral dissertation]. Faculty of Graduate Studies, University of Western Ontario. https://central.bac-lac.gc.ca/.item?id=NR39254&op=pdf&app=Library&oclc_number=665191412.

[20] Paap D, Schrier E, Dijkstra PU (2019). Development and validation of the working alliance inventory Dutch version for use in rehabilitation setting. Physiother Theory Pract.

[21] Leibert TW, Dunne-Bryant A (2015). Do common factors account for counseling outcome?. Journal of Counseling & Development.

[22] Flückiger C, Del Re AC, Wampold BE, Horvath AO (2018). The alliance in adult psychotherapy: a meta-analytic synthesis. Psychotherapy.

[23] Sturgiss EA, Rieger E, Haesler E (2019). Adaption and validation of the working alliance inventory for general practice: qualitative review and cross-sectional surveys. Fam Pract.

[24] Stewart MA (1984). What is a successful doctor—patient interview? A study of interactions and outcomes. Soc Sci Med.

[25] Stewart MA (1995). Effective physician—patient communication and health outcomes: a review. CMAJ.

[26] Stewart M (2005). Reflections on the doctor—patient relationship: from evidence and experience. Br J Gen Pract.

[27] Hermans L, Olde Hartman T, Dielissen PW (2018). Differences between GP perception of delivered empathy and patient-perceived empathy: a cross-sectional study in primary care. Br J Gen Pract.

[28] Burt J, Abel G, Elliott MN (2018). The evaluation of physicians' communication skills from multiple perspectives. Ann Fam Med.

[29] Mercer SW, Maxwell M, Heaney D, Watt GC (2004). The consultation and relational empathy (CARE) measure: development and preliminary validation and reliability of an empathy-based consultation process measure. Fam Pract.

[30] Britt H, Miller GC, Henderson J (2016). General practice activity in Australia 2015–16.

[31] Australian Bureau of Statistics (2018). Patient experiences in Australia 2017–18.

[32] Bikker AP, Fitzpatrick B, Murphy D, Mercer SW (2015). Measuring empathic, person-centred communication in primary care nurses: validity and reliability of the consultation and relational empathy (CARE) measure. BMC Fam Pract.

[33] Yong AG, Pearce S (2013). A beginner’s guide to factor analysis: focusing on exploratory factor analysis. Tutor Quant Methods Psychol.

[34] Osborne J, Costello AB (2004). Sample size and subject to item ratio in principal components analysis. Practical Assessment, Research and Evaluation.

[35] van der Steen JT, Kruse RL, Szafara KL (2008). Benefits and pitfalls of pooling datasets from comparable observational studies: combining US and Dutch nursing home studies. Palliat Med.

[36] Etz RS, Zyzanski SJ, Gonzalez MM (2019). A new comprehensive measure of high-value aspects of primary care. Ann Fam Med.

[37] Perdrix S, de Roten Y, Kolly S, Rossier J (2010). The psychometric properties of the WAI in a career counseling setting: comparison with a personal counseling sample. J Career Assess.

[38] Tatman AW, Love KM (2010). An offender version of the Working Alliance Inventory-short revised. J Offender Rehabil.

[39] Sturgiss EA, Sargent GM, Haesler E (2016). Therapeutic alliance and obesity management in primary care — a cross-sectional pilot using the Working Alliance Inventory. Clin Obes.

